# Dual inhibition of complement and Toll-like receptors as a novel approach to treat inflammatory diseases—C3 or C5 emerge together with CD14 as promising targets

**DOI:** 10.1189/jlb.3VMR0316-132R

**Published:** 2016-08-31

**Authors:** Andreas Barratt-Due, Søren Erik Pischke, Per H. Nilsson, Terje Espevik, Tom Eirik Mollnes

**Affiliations:** * Department of Immunology, Oslo University Hospital, and K. G. Jebsen Inflammation Research Centre, University of Oslo, Oslo, Norway;; † Department of Emergencies and Critical Care, Oslo University Hospital, Oslo, Norway;; ‡ Centre of Molecular Inflammation Research and Department of Cancer Research and Molecular Medicine, Norwegian University of Science and Technology, Trondheim, Norway;; § Research Laboratory Nordland Hospital, Bodø, Norway; and; ¶ K. G. Jebsen Thrombosis Research and Expertise Center, University of Tromsø, Tromsø, Norway

**Keywords:** innate immunity, inflammation, therapy

## Abstract

The host is protected by pattern recognition systems, including complement and TLRs, which are closely cross-talking. If improperly activated, these systems might induce tissue damage and disease. Inhibition of single downstream proinflammatory cytokines, such as TNF, IL-1β, and IL-6, have failed in clinical sepsis trials, which might not be unexpected, given the substantial amounts of mediators involved in the pathogenesis of this condition. Instead, we have put forward a hypothesis of inhibition at the recognition phase by “dual blockade” of bottleneck molecules of complement and TLRs. By acting upstream and broadly, the dual blockade could be beneficial in conditions with improper or uncontrolled innate immune activation threatening the host. Key bottleneck molecules in these systems that could be targets for inhibition are the central complement molecules C3 and C5 and the important CD14 molecule, which is a coreceptor for several TLRs, including TLR4 and TLR2. This review summarizes current knowledge of inhibition of complement and TLRs alone and in combination, in both sterile and nonsterile inflammatory processes, where activation of these systems is of crucial importance for tissue damage and disease. Thus, dual blockade might provide a general, broad-acting therapeutic regimen against a number of diseases where innate immunity is improperly activated.

## Abstract (precis)

Review of how targeting key upstream molecules at the recognition phase of innate immunity exert anti-inflammatory effects; a potential therapeutic regimen for inflammatory diseases.

## Introduction

The ingenious inflammatory network preserves host integrity and is essential in homeostasis. To fulfill host protection, the immune system needs sensors that respond to potential dangerous inducers, either of exogenous (PAMPs) or endogenous (DAMPs) origin. The instantly acting sensors of innate immunity are the first line of defense by recognizing these conserved molecular structures or patterns. PRRs represent an upstream immunologic hierarchy, sensing danger and subsequently signaling downstream through a multitude of different pathways. PRRs are classified based on their ligands and downstream effector pathways [1]. Nevertheless, pattern recognition may occur via several PRRs concurrently, thereby integrating different pathways in a balanced response via cross-talk. Under normal conditions, activation of PRRs induces local and self-limited effector responses that maintain homeostasis. However, improper or uncontrolled activation may induce local tissue damage or cause systemic imbalance, which may be fatal, as seen in SIRS and septic shock.

According to the danger model introduced by Matzinger [2], the immune system recognizes DAMPs, irrespective of their nature being exogenous microbes or endogenous “damaged self.” In principal, this suggests that different threats to the host unleash fairly similar initial host inflammatory responses. The Inflammation and Host Response to Injury Large-Scale Collaborative Research Program [3] underscored this important point by demonstrating the similarities in leukocyte gene expression patterns upon different severe injuries in humans. It showed that trauma, burn injury, and low-dose bacterial endotoxin infusion to humans induced a far more equal than different global reprioritization of the leukocyte gene expression, involving increased expression of proinflammatory and compensatory anti-inflammatory innate immune genes and decreased expression of genes involved in adaptive immunity. Thus, the targeting of key molecules belonging to the main systems of recognition has the propensity to act broadly, on numerous pathways, and can be an important tool in attenuating severe and harmful inflammation.

The complement system and TLRs are both important upstream components of the innate immune system, recognizing ligands of exogenous and endogenous origin initiating downstream inflammatory effects. These sensors and effector pathways act as partly individual branches but are connected through a considerable cross-talk, representing a vast redundancy of the host defense [4–7]. In 2008, we [8] postulated a hypothesis of combined blocking of key upstream molecules in the complement system and the TLR family as a therapeutic regimen for conditions with uncontrolled activation as a result of innate immune recognition. We have searched for upstream bottleneck molecules acting at the level of pattern recognition as targets for efficient inhibition of the inflammatory response.

The present review highlights the significance of complement and TLRs in various inflammatory models and importantly, demonstrates the efficacy of single and combined inhibition of these 2 upstream branches of innate immunity. The results comprise data from different experimental inflammatory ex vivo and in vivo models, including the use of different species. The interpretations of data from the large animal studies can be integrated into a complex context where dynamic relations among physiology, inflammation, thrombogenicity, and specific organs emerge.

## COMPLEMENT INHIBITION

The complement system was discovered in the 1890s, where Nuttal, Bordet, and others described it as a “lytic system” killing bacteria. The system was first named alexin, and Bordet introduced the term “complement” when he, in 1895, discovered that this was a heat-labile system present in normal human serum and was “complementing” the antibodies in killing the bacteria. Of interest, heat inactivation of serum (56°C for 30 min), which Bordet used to distinguish complement (heat labile) from antibodies (heat stable), is still a routine method used in our daily laboratory work in 2016—“heat-inactivated serum.” Notably, this procedure is by no means specific for complement activity, and caution should be taken when interpreting data from such sera [9].

Nearly 100 yr later—in 1984—there was a meeting of The Royal Society (London, United Kingdom) on complement, and the summary in *Immunology Today* (now *Trends in Immunology*) stated the following: “Many immunologists hold that complement is baffling or irrelevant or, most conveniently, both but a recent meeting emphasized that complement is interesting and that it may be important, even only as an elegant model system” [10]. It was, however, reasonable to ask how nature could have created such a molecularly fascinating system just for fun and entertainment for us. At that time, complement was simply regarded as a protective system against infections. During the last 30 yr, it has been a “complement revolution,” documenting complement to be involved in innumerable biologic systems contributing to host homeostasis, and today, complement inhibitors are in clinical use.

**Figure 1.**
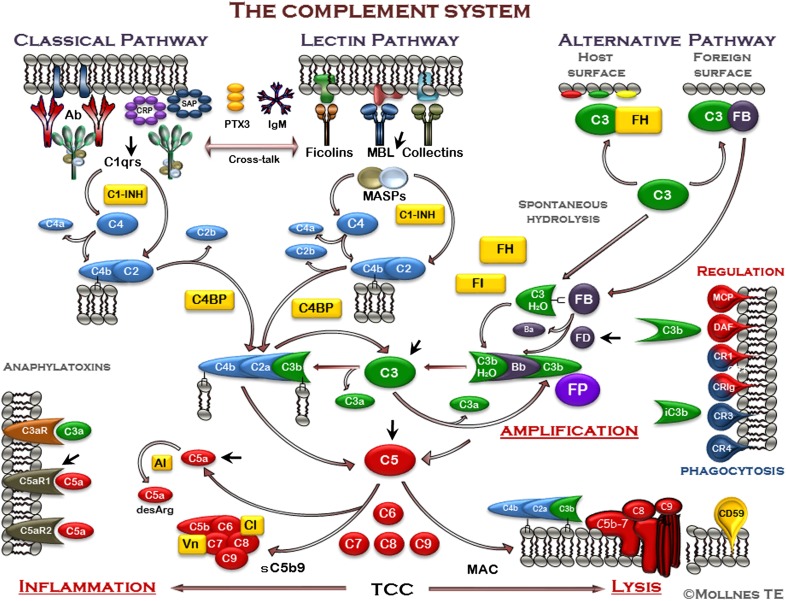
The complement system. The complement system can be activated through 3 pathways (top), all converging to the cleavage of C3 to generate C3a and C3b (middle). The classic pathway (CP) is typically activated by antibodies, but also, pentraxins (PTX), including C-reactive protein (CRP), serum amyloid P component (SAP), and PTX3 can activate C1q. The lectin pathway (LP) is activated through recognition of carbohydrates by mannose-binding lectin (MBL), ficolins, and collectins. Furthermore, LP activation may be mediated through IgM antibodies, e.g., directed against damaged self-antigens. The alternative pathway (AP) is activated by foreign or damaged own cells, facilitated by the continuous spontaneous hydrolysis of C3. AP also has an important function in the complement system, providing an amplification loop that enhances C3 activation independent of which pathway is initially activated. This effect is mainly a result of properdin (FP), the only positive regulator in the complement system, which stabilizes the C3 convertase. Activation of C3 leads to formation of a C5 convertase, cleaving C5 into C5a and C5b. The anaphylatoxins C3a and C5a bind to the receptors C3aR, C5aR1, and C5aR2, leading to downstream production of inflammatory mediators (bottom). C5b initiates the formation of the TCC, which forms the membrane attack complex (MAC) if inserted into a membrane (bottom). This may lead to lysis of bacteria and cells or in sublytic doses to activation of cells. The cleavage and inactivation of C3b generate iC3b, bind to complement receptors 3 (CR3; CD11b/CD18) and 4 (CR4; CD11c/CD18), facilitating phagocytosis, oxidative burst, and downstream inflammation (right). The complement system is tightly regulated by soluble inhibitors (yellow), including C1 inhibitor (C1-INH), factor H (FH), factor I (FI), C4-binding protein (C4BP), anaphylatoxin inhibitor (AI) inactivating the anafylatoxins (e.g., C5a to C5adesArg), vitronectin (Vn), and clusterin (Cl), keeping the continuous low-grade activation in the fluid phase in check. Host cell membranes are equipped with a number of inhibitors to protect them against attack by complement (right), including membrane cofactor protein (MCP; CD46), CR1 (CD35), decay accelerating factor (DAF; CD55), controlling C4 and C3 activation, and CD59 protecting against final assembly of the C5b-9 complex. Some attractive targets for therapeutic inhibition are indicated with black arrowheads, e.g., specific CP activation by one of the C1qrs components, specific LP activation by MBL and MASP intervention, and specific AP activation by neutralizing factor D (FD), which will attenuate the amplification of the system induced by all initial activation mechanisms. The inhibition of C3 is the broadest possible strategy, whereas inhibition of C5 cleavage will clock both the inflammatory potent C5a fragment and formation of the inflammatory and lytic C5b-9 complex. Alternatively, C5a can be inhibited, preserving the C5b-9 pathway, or the anaphylatoxin receptors can be blocked to prevent signaling. In particular, the blocking of C5aR1 will attenuate inflammation, whereas the effect of blocking C3aR and C5aR2 receptors is to be studied in more detail, as they might have more anti-inflammatory effects.

The complement system is built up of multiple proteins organized in 3 separated pathways for broad recognition, with a central C3 convertase for potent response amplification and an effector phase acting instantly upon cleavage of C3 and C5 (**Fig. 1**). Activation is tightly balanced by numbers of membrane and fluid-phase regulators targeting different levels in the complement cascade as recently reviewed in Bajic et al. [11].

To achieve a global inhibition of complement, C3 is a suitable protein to target, as all activation pathways converge at this step after having recognized danger. Furthermore, C3 is discriminated from self vs. nonself surfaces and is therefore an ultimate target for unspecific pattern recognition [12]. An important part of the mechanism of this discrimination is the competition between factors B and H for binding to C3b; i.e., C3b bound to a foreign surface favors binding of factor B with propagation of the activation, whereas on a self surface, factor H binds and inhibits further activation. Short-term inhibition of C3 would be of no concern, provided antibiotic prophylaxis, whereas long-term systemic inhibition would increase the risk of infection, as the main complement protection against infection is opsonization by C3 of microbes. The targeting of C5 is an alternative approach. Although this molecule belongs to the downstream part of the complement cascade, it is a definite upstream molecule in the inflammatory reaction [13]. The blocking of C5 prevents the liberation of the potent anaphylatoxin C5a and subsequent numerous proinflammatory effects [14]. Furthermore, C5 blockade prevents the formation of C5b that induces the assembly of C5b-9, which can lyse certain pathogens and cells but probably more important, in vivo can exert sublytic proinflammatory activity quite similar to that of C5a [15, 16].

The number of inflammatory diseases associated with complement-mediated pathophysiology is steadily increasing, as is the interest for complement-based therapy. Eculizumab was, in 2007, the first complement-targeting drug approved by the FDA for the treatment of PNH and later, also extended to include aHUS [17, 18]. Eculizumab is a mAb targeting C5, which together with C3, is one of the bottleneck molecules in the complement system and an obvious target for an efficient and general complement blockade. To minimize undesired effector functions related to the different IgG subclasses, eculizumab consists of sequences from both IgG2 and IgG4, substantially reducing FcγR binding (IgG2 portion) and complement activation (IgG4 portion; **Table 1**) [17]. However, the plethora of complement inhibitors in clinical trials targeting a number of different components gives an idea of the complexity of complement-driven inflammation, as previously reviewed [19–21]. Indications for complement-based therapeutics may cover acute systemic diseases to slow progressing or chronic organ-specific disorders [22]. The question arises not only which component to block but also, where, when, and how to block.

**TABLE 1.** Selected inhibitors of the complement system (A), the TLRs (B), and the combination thereof (C)

System	Name, substance	Target molecule, mode of action	Data from experimental and clinical studies	Reference
A	Compstatin, cyclic peptide (Cp40)	C3, prevents cleavage exclusively in primates	Evaluated in numerous preclinical studies, including sepsis, age-related macular degeneration, periodontal diseases, and transplantation	[28]
	Eculizumab, IgG2/4 mAb	C5, prevents cleavage in humans	Reduces intravascular hemolysis in PNH patients; inhibits complement-mediated TMA*^a^* and improves renal function	[99]
				[18]
	Coversin (OmCI), a tick-derived lipocalin protein	C5 and LTB4, prevents C5 cleavage, capture LTB	Ablates complement activity in several species; attenuates immune complex-induced acute lung injury in mice	[31]
				[32]
B	OPN-301 (mouse mAb) and OPN-305 (humanized IgG4 mAb)	TLR2, prevents dimerization with TLR1 and/or -6, inhibiting downstream cytokine production	Reduces infarction size and preserve cardiac function in pigs after I/R injury	[53]
	Eritoran tetrasodium (E5564), synthetic lipid A LPS analog	MD2/TLR4, prevents LPS-mediated NF-κB activation	Attenuates endotoxin-induced cytokine release in humans but did not reduce mortality in severe sepsis	[61]
				[62]
	IC-14, IgG1 mAb (18E12)	CD14 (coreceptor for several TLRs) prevents CD14 interaction with respective TLRs, blocking downstream signaling	Attenuates endotoxin-induced cytokine release in humans	[67]
	Anti-CD14, IgG2/4 mAb (r18D11)		Attenuates endotoxin-induced cytokine release and reduced Fc-mediated effects	[71]
	Anti-CD14 (pig), IgG2/4 mAb (rMIL2)		Attenuates *Escherichia coli*-induced cytokine release, avoiding undesired Fc-mediated effects	[71]
C	Anti-human C2 (IgG1, clone 175-26), anti-human D (IgG1, clone G3-519), and anti-human CD14 (clone 18D11)	C2, factor D (functional blocking of all pathways before the level of C3) and CD14	Meconium-induced inflammation (sterile) in whole blood was abolished by the dual blockade.	[96]
	Coversin and anti-mouse CD14 antibody, clone biG 53 F(ab′)_2_	C5 and CD14	Increased median survival (*P* = 0.001) in a model of polymicrobial sepsis in mice	[91]
	Compstatin, anti-human CD14 F(ab′)_2_ (clone 18D11), C5aR antagonist (PMX53)	C3, C5a receptor, and CD14	Microarray revealed 80% reduction in fold change in response when whole blood was challenged with *E. coli*.	[77]
	Coversin and anti-porcine CD14 IgG2/4 mAb (rMIL-2)	C5 and CD14	Improved survival and hemodynamic parameters in a model of porcine polymicrobial sepsis	[92]

a TMA, Thrombotic microangiopathy.

Drugs in clinical phase targeting complement are mainly found at 3 levels: 1) recognition phase, 2) C3/C3 convertase, and 3) C5/C5a/C5aR1. Molecular targets at the recognition phase include C1s and MASP-2, where blockade is investigated for the treatment of cold agglutinin disease and complement-mediated thrombotic microangiopathy, respectively [23]. Furthermore, concentrates of C1 inhibitor are tested in complement-related indications [24, 25]. However, C1 inhibitor is not a specific complement inhibitor but also targets serine proteases within the coagulation and contact system, as well as a number of nonprotease effects. Although it is an important regulator of the autoactivation of C1qrs [26], it is less efficient as a complement inhibitor under triggering conditions where high supraphysiological doses are needed for efficient inhibition [27].

The level of C3 activation encompasses drugs preventing C3 cleavage by direct-targeting C3. The cyclic peptide compstatin and later-developed acetylated derivatives, including Cp40, inhibit complement activation by binding C3, sterically preventing the binding of the convertase and C3 cleavage [28] (Table 1). It has been used in a wide range of different inflammatory disease models, effectively attenuating complement activation in humans and nonhuman primates, but it is still not available for clinical use. Another targeting approach at this level is to prevent the formation of the alternative pathway C3 convertase, in particular, by blocking the rate-limiting factor D. This level of inhibition also comprises several molecules that promote C3 regulation, either by exerting cofactor activity or convertase decay acceleration. Indications of interest for inhibition at the C3 level are, e.g., age-related macular degeneration and C3 glomerulopathy [29, 30]. The majority of drugs targeting complement activation is found at the level of C5/C5a/C5aR1. C5 is an attractive target, as generation of proinflammatory C5a and TCC are prevented, but the C3 and upstream effects are unaffected. In addition to eculizumab, there are several nonantibody-based molecules in clinical trials for inhibition of C5 cleavage. An interesting example is the tick-derived lipocain protein, OmCI, also known as coversin. This molecule binds to and prevents cleavage of C5, additionally inheriting an internal binding pocket capturing LTB4 [31, 32] (Table 1). The potent anaphylatoxin C5a exerts pronounced proinflammatory activity through the G-coupled receptor C5aR (CD88) and interacts through the non-G-coupled receptor C5L2, exerting both pro- and anti-inflammatory activity [33]. Molecules that specifically target the C5a–C5aR1 axis, directed against the anaphylatoxin or the receptor, are being tested in intractable human diseases [34]. Notably, indications for complement inhibition at the level of C5, beyond PNH and aHUS, have rapidly emerged during the last years, and promising experimental and preclinical results are found within a wide range of inflammatory diseases, such as myasthenia gravis, transplant-associated thrombotic microangiopathy, catastrophic anti-phospholipid syndrome, and neuromyelitis optica [35–38].

Clinical trials, registered in FDA and the European Medicines Agency, using complement-targeted therapeutics, study primarily inflammatory diseases with a clear complement-dependent profile, many caused by mutations in complement genes related to either activation by gain of function or regulation by loss of function, which both lead to an homeostatic imbalance, producing pathologic, increased complement activation [39]. It remains to be shown whether complement inhibition can be successful in more broadly triggered inflammation, such as in sepsis or I/R injuries, as seen, e.g., in the course of transplantation or polytrauma. In such a setting, the risk is that solely complement inhibition will be ineffective, as redundancy by other inflammatory systems may operate. Here, one might obtain an increased effect by simultaneously also targeting other routes of the inflammation, such as, e.g., the TLRs.

## TLR INHIBITION

**Figure 2.**
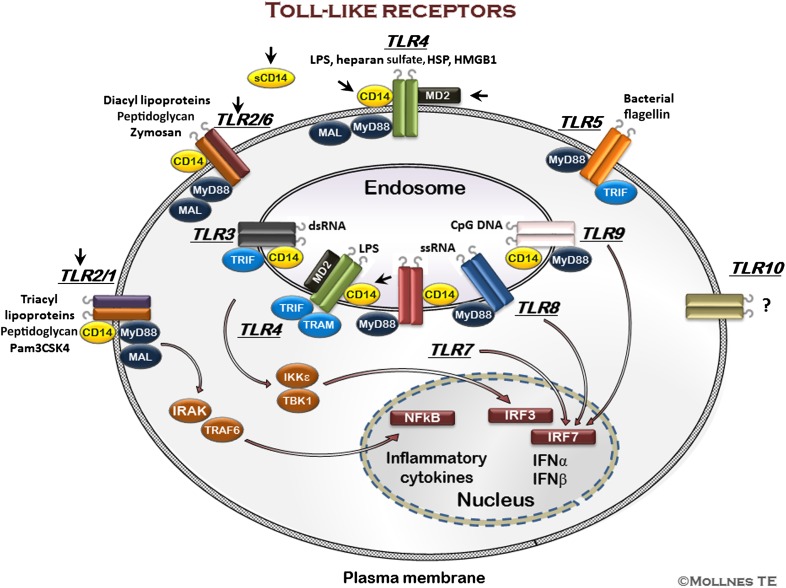
The TLRs. TLRs are transmembrane proteins recognizing conserved patterns of microbial structures, as well as damage self-molecules. Ten TLRs have been described in humans, the first 9 with defined ligands. TLR1, -2, -4, -5, and -6 are plasma membrane receptors, whereas TLR3, -7, -8, and -9 are intracellular, located to the endosomal membrane. TLR2 heterodimerizes with TLR1 or -6, whereas all others homodimerize. TLR4 is translocated from the plasma membrane, where it serves as the LPS receptor, with MD2 and CD14 as coreceptors, to the endosomal membrane. All TLRs, except TLR3, use MyD88 as one of their adaptor proteins. TLR2, -4, and -5 signal through IL-1R-associated kinases (IRAKs) and TNFR-associated factor 6 (TRAF6) to activate NF-κB to produce proinflammatory cytokines, whereas TLR7, -8, and -9 activate IRF7, and intracellular TLR4 activates IRF3 to produce IFN-α and -β. CD14 is a coreceptor for several of the TLRs. It has been known for years that TLR4 and TLR2 use CD14, but recently, CD14 has been described as a coreceptor, at least for mice, also for TLR3, -7, and -9. Some attractive targets for therapeutic inhibition are indicated with black arrowheads. Neutralization, usually using mAb, of both sCD14 and membrane-bound CD14 will inhibit LPS binding to TLR4. CD14 is a cofactor for a number of TLRs—TLR2 being the best documented—thus, CD14 acts as a potent molecule to target several TLR members. Specific TLR inhibitors, including the lipid A antagonist eritoran, blocks the TLR4/MD2 complex, and the humanized anti-TLR2 antibody prevents the dimerization of TLR2 with TLR1 and TLR6. A number of other specific inhibitors of both membrane-bound and intracellular TLRs and their signaling molecules are under development. HSP, heat shock protein; HMGB1, high mobility group box 1; MAL, MyD88 adapter-like; Pam3CSK4, palmitoyl-3-cysteine-serine-lysine-4; TBK1, TANK-binding kinase 1; IKKɛ, IκB kinase ɛ.

Probably, the best described PRRs are the TLRs, which are up-regulated upon stimulation and found on nearly all cells, especially on cells important in innate immunity signaling, such as dendritic cells, leukocytes, macrophages, and endothelial cells. Ten different human TLRs are known today (TLR1–10), whereas there are 13 known in mice [40, 41]. TLRs are transmembrane proteins, which recognize distinct PAMPs [40] and DAMPs [41] (**Fig. 2**). TLR1, -2, -4, -5, -6, and -10 are located in the plasma membrane, whereas TLR3, -7, -8, and -9 are found intracellularly on endosomal membranes. All TLRs, except TLR3, use MyD88 as an adapter molecule to induce signaling. TLR4 has a unique role, as it initiates 2 different signaling pathways. From the plasma membrane, TLR4 mediates MyD88-dependent signaling, leading to rapid NF-κB activation and production of proinflammatory cytokines. Moreover, TLR4 can translocate to endosomes and phagosomes, where it activates a TRAM–TRIF-dependent pathway, leading to IRF3 phosphorylation and IFN-β production. Both CD14 and Rab11a control translocation of TLR4 to endosomes and phagosomes [42, 43]. The TLRs are dependent on interaction with a broad range of extracellular accessory proteins and cofactors to elucidate full-scale intracellular signaling and induction of proinflammatory gene transcription and cytokine release. Numerous accessory or coreceptor molecules are known and putative targets for therapy. MD2 (LY96) is a 160-aa soluble protein, which is essential for LPS-mediated TLR4 signaling, as it binds the lipid A part of LPS and leads to TLR4 dimerization [44].

It is well established that CD14 enhances LPS responsiveness by binding LPS and facilitating LPS transfer to TLR4–MD2 [45]. In addition to TLR4, CD14 is important for TLR2 [46, 47]. CD14 is a 375-aa glycoprotein, comprising multiple leucine-rich repeats, and is present both as a membrane and soluble form (sCD14). CD14 can bind multiple PAMPs and DAMPs, including LPS, peptidoglycan, polyinosinic:polycytidylic acid, and DNA. Once bound, CD14 chaperones these pathogenic molecules to the correct TLR, and today, CD14 has been shown to be a coreceptor, not only for TLR2 and TLR4 but also, at least for mice, for TLR3, -7, and -9 [46, 48]. CD14 is also implicated in LPS signaling through non-TLRs, such as the purinergic P2X7 receptor for ATP [49]. Thus, CD14 is of utmost interest as a bottleneck-target goal at the recognition phase of innate immunity.

Several inhibitors, including antibodies or compounds against TLRs and coreceptors, such as MD2 and CD14, have been characterized. However, most of them have only been evaluated in vitro and in rodent models, which makes it difficult to assess their clinical relevance. The main findings from these studies are that TLR inhibition is beneficial in viral infections, SIRS, and sepsis, as well as in cerebral, myocardial, renal, and hepatic I/R injury, but may be detrimental in intestinal I/R injury [40, 50, 51].

TLR inhibition is only sparsely evaluated in large animal models and clinical studies. TLR2 receptor blockade has been evaluated in mice with the mAb OPN-301, which prevents TLR2 dimerization with TLR1 or TLR6 and downstream cytokine production [52] (Table 1). TLR2 inhibition by the humanized IgG4 OPN-305 reduced infarction in a pig model of myocardial I/R injury [53] and was well tolerated by healthy human volunteers in a phase I clinical study [54]. Currently, a phase II study with OPN-305 is starting, with the aim to treat kidney transplant patients at high risk to develop delayed renal graft function (see clinicaltrials.gov: NCT01794663).

Specific inhibition of TLR4 has been tried using several small molecular agents. The small compound TAK-242 (resatorvid) is a direct TLR4 inhibitor, which blocks the intracellular binding of coadaptor proteins to TLR4 and thus, inhibits downstream signaling [55]. Pretreatment with TAK-242 increased survival in LPS-challenged guinea pigs [56], prevented systemic circulatory deterioration, and reduced acute kidney injury in *E. coli* infused sheep [57], also when administered 12 h after onset of challenge [58]. However, TAK-242 failed in a phase III study to reduce IL-6 concentration in septic patients with either shock or respiratory failure, and it had no significant effect on mortality [59].

Another TLR4 inhibitor, E5564 (eritoran), is a structural analog of the lipid A portion of LPS, which binds to MD2 and thus, prevents LPS binding and TLR4 activation [60] (Table 1). Eritoran eliminated all clinical effects and decreased IL-6 and TNF concentrations in healthy humans subjected to LPS challenge [61]. Although promising in a phase II study, no beneficial effects could be demonstrated in a large (*n* = 1961) phase III study in severe septic patients [62]. Eritoran has been shown to protect mice from influenza infection and was also shown to interact with CD14 [63]. Recently, eritoran was compared with a neutralizing anti-CD14 antibody in a human whole-blood model with respect to effect on Gram-negative and -positive, bacteria-induced inflammation [64]. In line with previous findings, eritoran and anti-CD14 mostly inhibited Gram-negative-induced inflammation, whereas Gram-positive inflammation was more complement dependent, possibly explaining the lack of effects of eritoran in a broad sepsis population. Additionally, anti-CD14 was more efficient than eritoran, in particular, with respect to monocyte responses. When combined with a complement inhibitor, anti-CD14 was also more efficient in attenuating the inflammatory responses than eritoran, underscoring CD14 as a more broad-acting recognition molecule.

As mentioned above, CD14 is implicated in activation of several TLRs. It has been shown that a blockade of CD14 attenuates central inflammatory cytokines in plasma and organs and reduces the thrombogeneic state induced by *E. coli* sepsis in pigs [65, 66]. In humans challenged with LPS, CD14 blockade has been evaluated by IC-14, a chimeric mAb, demonstrating reductions of inflammatory plasma cytokines [67, 68] (Table 1) and a possible link of CD14 to the coagulation cascade [69]. However, phase II studies in septic patients (*n* = 40) revealed inconclusive results, with 1 patient experiencing anaphylaxis upon IC-14 infusion [70], whereas a study in patients with acquired pneumonia was completed in 2005 without publication of results (see clinicaltrials.gov: NCT00042588). No further studies using IC-14 are currently registered. Our group has used mouse CD14 mAb to produce recombinant chimeric variants on a human IgG2/IgG4 backbone, which lack detrimental Fc-mediated effects for both pig (rMIL-2) in vitro and in vivo and human (r18D11) in vitro studies [71] (Table 1).

Taken together, TLR inhibition reduces a variety of proinflammatory cytokines and has even shown some promising clinical effects. Nevertheless, in all studies, a significant inflammatory activation was still present, consistent with redundancy by activation of other PRRs, including the complement system.

## COMBINED INHIBITION OF COMPLEMENT AND CD14

To dissect to which degree complement was responsible for the downstream inflammatory network, we developed a blood model to investigate the mutual interaction among all inflammatory systems in human whole blood [72]. The use of anticoagulant was critically important, as standard anticoagulation interferes with most inflammatory systems, including complement. The blood was anticoagulated with the highly specific recombinant form of the thrombin inhibitor hirudin (lepirudin), which does not interfere with complement and the other inflammatory systems but has the limitation that effects of thrombin cannot be investigated. To our knowledge, this model is the human whole blood ex vivo model closely mimicking the physiologic condition.

With the use of complement inhibitors in this model, it was shown that certain bacterial-induced inflammatory responses were virtually, totally dependent on complement, such as granulocyte CD11b expression and oxidative burst. The complement inhibitory effect on cytokines varied, and only a few of them were substantially, demonstrating a vast inhibitory effect on IL-8, whereas IL-6 remained partly unaffected in Gram-negative-induced inflammation [73]. Thus, this model, to a great extent, answered the questions on which readouts were mainly complement dependent and which were not.

It was of utmost importance to find an additional inhibitor that could enhance the anti-inflammatory effect of complement inhibition, and we hypothesized that CD14 could be another bottleneck target to block, as CD14 is a central coreceptor in the TLR system. In 2007, the potency of combined inhibition of complement and TLRs in the whole-blood model was described for the first time, using Gram-negative-induced inflammation [74]. Single inhibition with C5a and CD14 reduced up-regulation of CD11b on monocytes and granulocytes only partly, whereas the combined inhibition completely abrogated the expression in both cell types.

### Combined inhibition in Gram-positive- and -negative-induced inflammation

*E. coli*-induced inflammation in human whole blood significantly increases the formation of a vast range of inflammatory mediators, including proinflammatory cytokines (TNF, IL-6, and IL-1β), chemokines (IL-8, MCP-1, MIP-1α, eotaxin, and IFN-γ-inducible protein 10), growth factors (vascular endothelial growth factor, basic fibroblast growth factor, G-CSF, and GM-CSF), and other ILs (IL-9, IL-15, and IL-17) [75]. Surprisingly, all mediators were abolished by a combined inhibition of CD14 and complement using a anti-C2 mAb and anti-factor D, which together, blocks all 3 initial complement pathways at the level before C3. A similar effect on the cytokine response was obtained by a combined inhibition of C3 and CD14 in Neisseria *meningitidis*-induced inflammation [76].

Although the CD14-driven pathway apparently plays a major role in Gram-negative-induced inflammation, complement plays a comprehensive part too. The complement dependence (C3 and C5) of a vast range of inflammatory responses, as well as the relative importance of CD14 in Gram-negative-induced inflammation, was delineated in blood of a C5-deficient human, nature’s own gene knockout model [73]. It was found that the release of cytokines and chemokines was, in general, more dependent on CD14 but in a varying degree, also complement dependent and some of them even inversely dependent on complement; i.e., inhibition increased their release. Expression of tissue factor, adhesion molecules, and oxidative burst was highly dependent on C5, whereas granulocyte–enzyme release was primarily dependent on C3. In general, granulocyte responses were mainly complement dependent, whereas monocyte responses were primarily dependent on CD14. Importantly, all studied inflammatory responses, including those inversely dependent on complement, were abolished by a combined inhibition of complement and CD14.

On a transcriptional level, microarray technology revealed a significant change—up- or down-regulation—in >2200 transcripts when human whole blood was challenged with *E. coli* [77]. As many as 70% of the genes were reversed by an average of 80% reduction in fold change in response to the dual inhibition of complement and CD14, in accordance with the pronounced effect of the inflammatory response observed in blood. This study underscores the presence of a fundamental crosstalk between complement and CD14. Synergistic effects were primarily observed, but also, different counteracting effects were present, i.e., complement counteracting the inhibitory effect of anti-CD14 or vice versa, confirming the vast interplay between these 2 branches of innate immunity.

**Figure 3.**
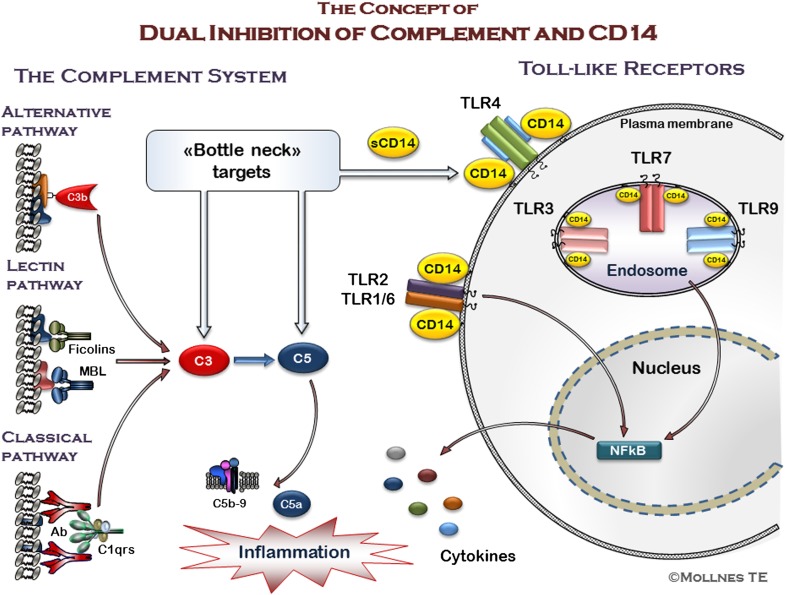
The “double blockade” of bottleneck recognition molecules at innate immune recognition. An upstream approach for inhibition of inflammation achieved by targeting the key complement molecules C3 or C5 and the CD14 molecule of the TLR family are proposed. Activation through all initial complement pathways converges at C3 and C5, and blocking of the bottleneck molecule C5 inhibits formation of the potent anaphylatoxin C5a, which is a main contributor in the pathogenesis of a number of disease conditions. As CD14 serves as a coreceptor for several of the TLRs, including the important TLR4 and TLR2, it might be regarded as a bottleneck molecule in the TLR family. Combined inhibition of these molecules will reduce the downstream inflammatory response substantially.

The effect of complement and CD14 inhibition on Gram-positive bacteria is sparsely evaluated. One study using various strains of Staphylococcus *aureus* in a human whole-blood model demonstrated that simultaneous inhibition of CD14 and complement efficiently reduced the inflammatory response [78]. However, and contrary to Gram-negative-induced inflammation, the responses were primarily dependent on complement, whereas CD14 played a lesser important role. However, the combined inhibition was more efficient toward the bacterial induced responses than either complement or CD14 inhibition alone and attenuated the responses similar to what was seen for the Gram-negative bacteria. A schematic presentation of the dual inhibition is presented in **Fig. 3**.

### Combined inhibition in experimental models of sepsis

The pathophysiology of sepsis is complex but can be regarded as a prototype of a host-threatening systemic inflammatory response unleashed by infection. For years, researchers have sought various inhibitory targets to attenuate and get control of the overwhelming and counterproductive sepsis-induced inflammatory response. Clinical studies using mAb toward the central proinflammatory cytokines TNF and IL-1β have failed to improve survival [79, 80]. Drotrecogin α (activated recombinant protein C) was initially thought to improve survival but was withdrawn from the marked a few years ago as a result of lack of efficacy [81]. In fact, all clinical immunomodulatory interventions toward sepsis have, in general, been disappointing [82].

Pigs are suitable for medical research, as they have closer anatomic, physiologic, and immunologic relations to humans compared with rodents [83], and additionally, enable repeated blood sampling for dynamic and comprehensive analysis. We have explored the field of sepsis by using pigs and developed models of Gram-negative sepsis induced by an incremental infusion of either *E. coli* [84] or *N. meningitidis* [85]. Both of these models induced hemodynamic alterations, capillary leak, and inflammatory responses corresponding to human pathophysiology and mimic, to a large extent, the initial phase of human septic shock. In *E. coli*-induced sepsis in pigs, combined inhibition of C5 and CD14 essentially abolished the formation of all proinflammatory cytokines, extensively inhibited up-regulation of CD11b on granulocytes, and partly prevented the procoagulant state of sepsis [86]. In addition, tissue samples from lung, heart, kidney, liver, and spleen were examined, all displaying a significantly reduced organ inflammatory load [87]. The substantial beneficial effect observed on the sepsis-induced thrombogenicity is of clinical importance, as thrombus formation is a key feature in sepsis that may be accompanied with impaired microcirculation and increased risk of organ dysfunction. Expression of tissue factor, the key initiator of coagulation in vivo, as well as formation of thrombin–antithrombin complexes, were profoundly attenuated by the combined regimen. These data are in accordance with the well-known cross-talk between complement and coagulation [88, 89], but notably, an additional effect of anti-CD14 was observed on the hemostatic parameters. Thus, it is likely that tissue factor expression was attenuated as a result of anti-C5 treatment but also reduced by the effect of anti-CD14 or the combination thereof [90].

The efficacy of the combined regimen is convincing also in polymicrobial sepsis, a clinically, more relevant model, mimicking abdominal sepsis seen after bowel rupture and anastomotic leak. In mice, combined inhibition of C5 and CD14 profoundly attenuated all inflammatory markers and importantly, significantly increased survival, whereas single inhibition of C5 or CD14 did not [91]. Comparable results were observed in pigs [92]. Dual inhibition of C5 and CD14 improved survival, was reflected by a significantly decreased plasma sC5b-9 level in treated animals, and correlated significantly with mortality.

The different sepsis studies demonstrate that inhibition of bottleneck molecules belonging to 2 main systems of recognition has the potential to act broadly on the numerous biomarkers and reveal pronounced effects on downstream mediators and importantly, on clinical outcome. Although the administration of inhibitors before the induction of sepsis is a limitation with these experiments, all studies clearly demonstrate the efficacy of upstream inhibition and belong to the field of science exploring the proof of concepts. In general, the timing of intervention is a great challenge within the field of sepsis; how can we properly identify the patient and provide treatment early enough and not at the time often described as the point of no return? With consideration of the latter, it is therefore interesting and promising that combined postchallenge inhibition of complement and CD14 significantly attenuate central proinflammatory biomarkers in experimental *E. coli*-induced inflammation ex vivo [93].

### Combined inhibition of endogenous-induced inflammation—the future

Today, no studies in big or small animals are published that evaluate combined TLR and complement inhibition in sterile inflammation. Therefore, an extensive area of research needs attention. Meconium, aspirated by term newborns, may induce a life-threatening respiratory condition called meconium aspiration syndrome [94]. Experimental studies demonstrate that piglets undergoing meconium aspiration syndrome have a massive increase in complement activation and cytokine release consistent with SIRS [95]. Sterile and endotoxin-free meconium incubated in cord blood and adult blood induces a vast inflammatory response that is almost abolished by a combined inhibition of complement and CD14 [96]. Thus, we are currently exploring this field by conducting an experimental, interventional study in newborn piglets using the combined treatment regimen.

Both complement and the TLRs are implicated in I/R injury, events that frequently happen in conjunction with infarction, trauma, and transplantation [97, 98]. An episode of I/R will lead to exposure of DAMPs from damaged tissue and activate innate immunity, including complement and TLRs. Ongoing studies from our group attempt to elucidate the efficacy of the combined regimen in different ex vivo and in vivo studies within this pathophysiological field. From a clinical point of view, such models will have a great translational value; thus, inhibitors can be administered before the detrimental reperfusion phase. In the case of a myocardial infarction, restoration of blood to the ischemic area is priority number one, but there is an interventional time window where inhibitors of innate immunity can be provided before reperfusion. During transplantation, therapy can be given from the time of organ harvesting to implantation.

## CONCLUSION

The targeting of bottleneck molecules at the level of innate immune recognition of PAMPs and DAMPs may present a particularly efficient way of attenuating the inflammatory response, as all known innate immune systems closely interact. Complement component C3 or preferentially, C5 inhibition, combined with neutralization of the CD14 molecule, fulfills this level of ambition. We suggest that this combined inhibition approach can be a clinically relevant treatment regimen to block an inappropriate and overwhelming inflammatory reaction and restore the homeostasis of the host.

## AUTHORSHIP

A.B.-D., S.E.P., P.H.N., T.E., and T.E.M. wrote the review.

## DISCLOSURES

The authors declare no conflicts of interest.

## Abbreviations

aHUS: atypical hemolytic–uremic syndrome
DAMP: damage-associated molecular pattern
FDA: U.S. Food and Drug Administration
I/R: ischemia/reperfusion
IRF: IFN regulatory factor
LTB4: leukotriene B4
MASP: mannose-binding associated serine protease
MD2: myeloid differentiation protein 2
OmCI: *Ornithodoros moubata* complement inhibitor
PAMP: pathogen-associated molecular pattern
PNH: paroxysmal nocturnal hemoglobinurea
PRR: pattern recognition receptor
rMIL-2: recombinant murine MIL-2
s: soluble
SIRS: systemic inflammatory response syndrome
TCC: terminal C5b-9 complement complex
TRAM: Toll/IL-1R domain-containing adapter-inducing IFN-β-related adaptor molecule
TRIF: Toll/IL-1R domain-containing adapter-inducing IFN-β
